# Quantitative Lipidomics Reveals Dynamic Lipid Profiles in *Cinnamomum camphora* Seed Kernels at Different Developmental Stages

**DOI:** 10.3390/plants15121887

**Published:** 2026-06-17

**Authors:** Han Xie, Yongjie Zheng, Yueting Zhang, Chenglin Luo, Ting Zhang, Wanwen Yu, Xuhui Zhang, Xinliang Liu

**Affiliations:** 1 National Key Laboratory for Development and Utilization of Forest Food Resources, Co-Innovation Center for Sustainable Forestry in Southern China, Nanjing Forestry University, Nanjing 210037, China; 15623286293@njfu.edu.cn (H.X.); l18359088807@163.com (C.L.); youeryuww@163.com (W.Y.); 2 Jiangxi Provincial Key Laboratory of Improved Variety Breeding and Efficient Utilization of Native Tree Species, Jiangxi Academy of Forestry, Nanchang 330032, China; zyj_bio2015@163.com (Y.Z.); yuetingzhang918@163.com (Y.Z.); zhangtycx@163.com (T.Z.)

**Keywords:** *Cinnamomum camphora*, kernels, quantitative lipidomics, developmental stages, triacylglycerol (TG), medium-chain fatty acids

## Abstract

*Cinnamomum camphora* seed kernels are a potentially valuable fatty oil resource; however, their lipid composition and dynamic changes during development remain poorly understood. In this study, morphological and anatomical observations combined with liquid chromatography–tandem mass spectrometry (LC-MS/MS)-based lipidomics were used to investigate lipid accumulation patterns in *C. camphora* seed kernels across five developmental stages. The results showed that seed development followed a distinctive pattern in which morphological maturation preceded physiological maturity. A total of 627 lipid molecules were identified and classified into 27 subclasses and 5 major classes. Among them, glycerolipids (GLs) and glycerophospholipids (GPs) were the dominant lipid classes, with triacylglycerols (TGs) representing the principal storage lipids. Approximately 84.2% of the detected lipids were unsaturated, indicating a highly unsaturated lipid profile. The fatty acid composition was enriched in medium-chain fatty acids (MCFAs), especially decanoic acid and lauric acid, suggesting that *C. camphora* seed kernel oil possesses distinctive compositional characteristics compared with conventional fatty oils. In addition, coenzyme Q (CoQ) showed relatively high abundance and dynamic accumulation during seed development. Differential lipid analysis further revealed that lipid remodelling occurred mainly during the early developmental stages and was significantly associated with glycerolipid and glycerophospholipid metabolism. Diacylglycerols (DGs), phosphatidylcholines (PCs), and phosphatidylethanolamines (PEs) decreased during early development, whereas TGs accumulated continuously from the middle stage onwards. Overall, this study provides a systematic characterisation of lipid composition and developmental dynamics in *C. camphora* seed kernels and offers a theoretical basis for their future utilisation as a novel functional fatty oil resource.

## 1. Introduction

*Cinnamomum camphora* is an evergreen tree species belonging to the family Lauraceae and the genus *Cinnamomum* [1]. Its seed kernel represents a distinctive fatty oil resource with considerable development potential. Previous studies have shown that *C. camphora* seeds have promising applications in medicine, the development of novel oils, and functional food research [2,3,4]. However, despite the growing market demand for functional edible oils derived from *C. camphora* seeds, efficient exploitation of this resource remains constrained by substantial germplasm heterogeneity, the long growth cycle of the species, and pronounced differences in lipid profiles among germplasms [5]. To date, most studies have focused on extraction processes and the bioactivities of *C. camphora* seed kernel oil [6,7,8], whereas comprehensive characterisation of its lipid composition remains limited.

*C. camphora* produces abundant seeds annually in China, with the annual yield of seeds exceeding one million tons. The oil content of its seed kernel ranges from 46.29% to 62.08%, with an average value higher than 50%. Accordingly, *C. camphora* seed is regarded as a widely available, low-cost and renewable fatty oil resource [2]. The oil is characterised by a high proportion of medium-chain triacylglycerols (MCTs) and is rich in caprylic acid (C8:0), capric acid (C10:0), lauric acid (C12:0), and myristic acid (C14:0) [9,10]. Compared with long-chain fatty acids, these fatty acids are more readily absorbed and metabolised and can provide energy more efficiently [11]. Therefore, they have attracted considerable attention because of their potential roles in regulating glucose and lipid metabolism, limiting fat accumulation, and supporting the development of value-added functional oils [7,12,13,14]. Moreover, *C. camphora* seed kernel oil has been reported to be safe and non-toxic, and the extracted oil products comply with relevant food and medicinal standards, highlighting its promise as a novel functional oil [15,16].

Lipids constitute a diverse class of organic compounds that are insoluble in water but readily soluble in non-polar organic solvents. They are indispensable for nutrient transport, energy conversion, signal transduction, and metabolic regulation, and are therefore essential to human health [17,18]. According to the LIPID MAPS classification system, lipids can be broadly divided into eight categories: fatty acids (FAs), glycerolipids (GLs), glycerophospholipids (GPs), sphingolipids (SPs), sterol lipids (STs), saccharolipids (SLs), prenol lipids (PRs) and polyketides (PKs) [19,20]. Many lipid species, especially GLs, consist of fatty acyl chains esterified to a glycerol backbone [21]. In fatty oilseed crops, the timing and extent of lipid accumulation, together with compositional remodelling during kernel development, are major determinants of oil quality and value.

Lipidomics has become an important approach for comprehensive analysis of lipid composition, identification of differentially abundant lipid species, and elucidation of lipid metabolic regulation. It has been widely applied in studies of oilseed crop quality [22]. Among available analytical platforms, mass spectrometry (MS) has been considered the central role in lipidomics because of its high sensitivity, broad dynamic range, and high resolution. In particular, liquid chromatography–tandem mass spectrometry (LC-MS/MS) enables efficient separation and reliable identification of complex lipid mixtures and is therefore widely used in plant lipidomics studies [23,24]. Previous studies have examined regional variations in the fatty acid composition of *C. camphora* seed kernels [2]. Lipidomic profiles have been reported for several other oil-rich biological materials, including *Glycine max* [25], *Arachis hypogaea* [26], *prunus persica* [27], *Carya illinoinensis* [28], and *Ophiocordyceps sinensis* [29]. However, the lipid composition of *C. camphora* seed kernels has not yet been systematically characterised, particularly with respect to dynamic lipid remodelling during development.

Seed lipid metabolism is a highly dynamic and orderly regulated process throughout seed development [30]. Continuous biosynthesis, accumulation, and structural remodelling of lipids profoundly determine the physical quality, nutritional properties, and industrial processing performance of mature seed oil. Developmental stage-specific alterations in lipid abundance, molecular species composition, and fatty acid distribution govern the overall oil characteristics, among which the temporal accumulation of TG, progressive enrichment of medium-chain fatty acids (MCFAs), and metabolic transition from membrane lipid synthesis to storage lipid accumulation represent critical physiological events supporting seed maturation [31,32]. Therefore, dissecting the dynamic patterns of lipid remodelling during seed development is essential to clarify the physiological basis of oil formation, and provides a theoretical foundation for optimal harvest timing, germplasm utilisation, and targeted improvement of functional oil traits in woody oil crops. Compared with conventional oilseed plants, *C. camphora* exhibits a unique asynchronous developmental characteristic, in which seed morphological maturation occurs earlier than physiological maturation, accompanied by distinct lipid metabolic reprogramming. Notably, *C. camphora* seed kernels are rich in MCFAs and CoQ, endowing its seed oil with superior nutritional and functional characteristics. Nevertheless, the dynamic alterations and regulatory mechanisms of lipid metabolism during *C. camphora* kernel development remain poorly understood, and a systematic lipidomic landscape covering the entire developmental period is still lacking. Therefore, this study investigated the developmental progression of *C. camphora* seed kernels. LC-MS/MS-based lipidomics was employed to characterise lipid composition and dynamic patterns of lipid accumulation across developmental stages, thereby providing a theoretical basis for elucidating oil formation in *C. camphora* seeds and supporting the development of novel functional oils.

## 2. Results

### 2.1. Morphological and Anatomical Observations of C. camphora Seeds at Different Developmental Stages

To clarify the pattern of lipid accumulation during *C. camphora* seed development, seeds were collected from late April to late October for morphological and anatomical observations. The fruit of *C. camphora* consists of a pericarp and a seed, and on the basis of fruit developmental characteristics, seed development was divided into five stages: S1, young fruit stage (7–32 days after flowering, DAF); S2, fruit expansion stage (32–81 DAF); S3, kernel formation stage (81–102 DAF); S4, kernel maturation stage (102–171 DAF); and S5, fruit ripening stage (≥171 DAF).

Following pollination in late April, the ovary enlarged and young fruits became visible by the end of April. At S1, the exocarp was green, the mesocarp was brownish, and the endocarp was white. During S2 (late May to mid-July), the fruit expanded rapidly, and the pericarp changed from light green to a deeper green as development progressed. During this period, the seed coat formed and adhered closely to the endocarp, the internal cavity gradually enlarged, and the endocarp hardened. Similarly, both the transverse and longitudinal diameters of the seed kernel increased markedly, with the longitudinal diameter consistently exceeding the transverse diameter. The transverse diameter increased from approximately 1 mm to 4 mm, whereas the longitudinal diameter increased from approximately 2 mm to 4 mm.

During S3 (mid-July to early August), the seed kernel underwent a further but modest increase in size, reaching average transverse and longitudinal diameters of 4.46 mm and 4.73 mm, respectively. At this stage, the boundaries among the exocarp, mesocarp, and endocarp became distinct, the cavity contained a transparent viscous liquid, and embryo initiation as well as cotyledon differentiation became evident. After mid-July, fruit enlargement ceased and the basic fruit shape became fixed. The endocarp gradually turned brown, hardened, and thickened, while the transparent liquid in the cavity gradually changed into a transparent gel-like substance.

**Figure 1:**
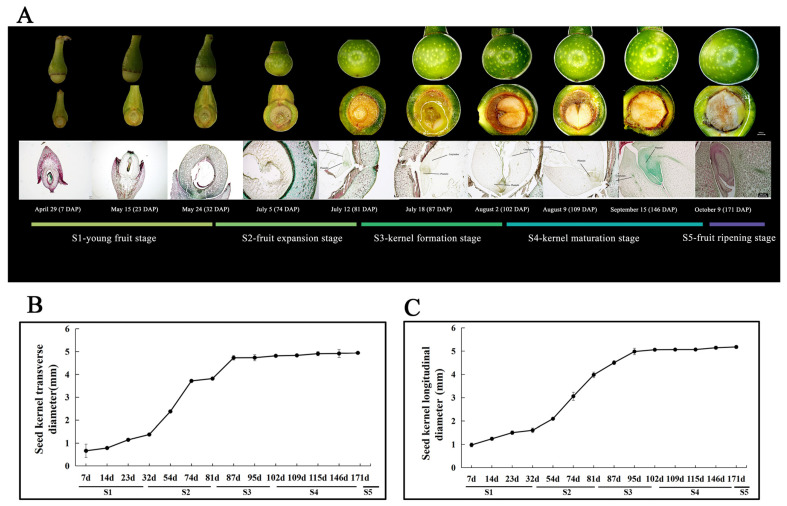
Morphological characteristics and changes in seed kernel traits during the development and ripening of *C. camphora* seeds. (**A**) Representative phenotypes of *C. camphora* seeds at different developmental stages. The upper panels show external morphology, and the lower panels show kernel cross-sections. (**B**) Transverse diameter of seed kernels. (**C**) Longitudinal diameter of seed kernels.

During S4 (early August to early October), especially after mid-August, the cotyledons developed from small butterfly-shaped lobes into a hemispherical structure that progressively filled the cavity, while the embryonic axis elongated. By S5 (mid-October onward), seed growth entered a relatively stable stage. At this stage, fruit colouration began, although the process was uneven among fruits, and individual fruits could turn completely blue-black within one week. Kernel size remained relatively stable, with average transverse and longitudinal diameters of 5.02 mm and 5.16 mm, respectively. Meanwhile, the exocarp became plump, the mesocarp gradually hardened, and the seeds reached full maturity before fruit abscission (Figure 1A–C).

### 2.2. Lipidomic Analysis of C. camphora Seed Kernels

#### 2.2.1. Qualitative Analysis of Lipids in *C. camphora* Seed Kernels

The quality of the lipidomic data was first evaluated using QC samples in both negative and positive modes. The overlapped total ion chromatograms (TICs) of all QC samples showed good spectral overlap, with stable retention times and consistent peak intensities (Figure S1A,B), confirming that the LC-MS system was well calibrated and stable during analysis. Most lipids were effectively separated within 12–17 min (Figure S1).

To investigate the lipid characteristics of *C. camphora* seed kernels, LC-MS/MS-based lipidomics was performed across five developmental stages. In total, 627 lipid molecules were identified and classified into five major categories, including 377 glycerolipids (GLs), 169 glycerophospholipids (GPs), 56 sphingolipids (SPs), 3 prenol lipids (PRs), and 22 fatty acyls (FAs) (Table S1). These lipids were further assigned to 27 subclasses, indicating substantial structural diversity and compositional complexity in *C. camphora* seed kernels.

**Figure 2:**
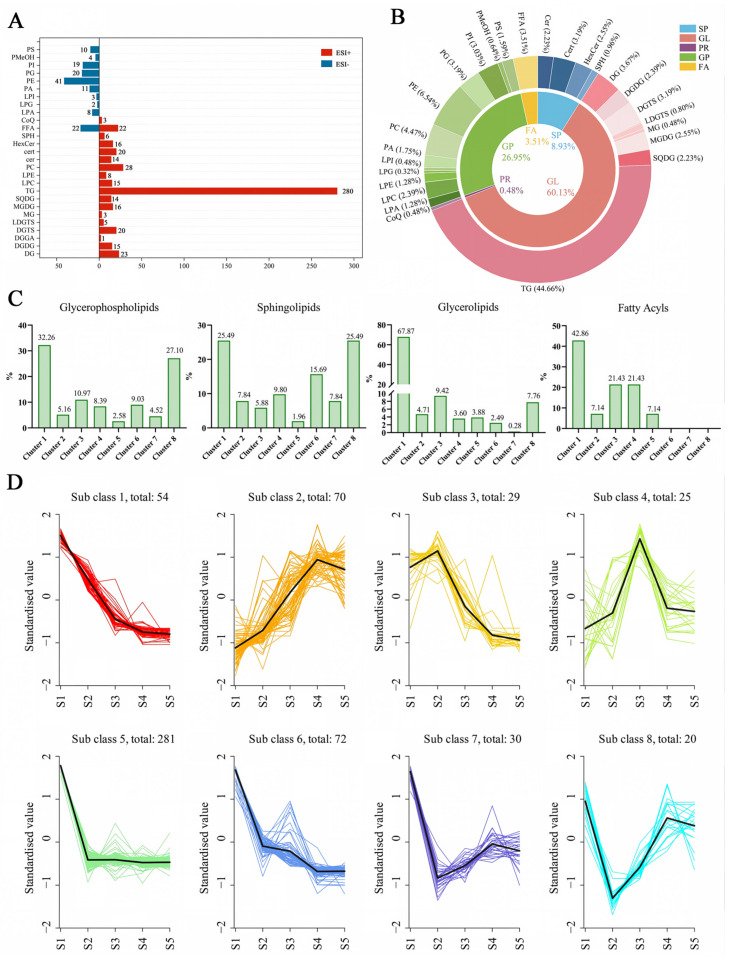
Lipid subclasses and dynamic changes in lipid molecules in *C. camphora* seed kernels. (**A**) Numbers of lipids identified in positive and negative ion modes. (**B**) Proportional distribution of lipid subclasses. (**C**) Relative proportions of GPs, SPs, GLs, and FAs across the eight clusters. (**D**) K-means clustering analysis of all identified lipid molecules. Colored lines represent individual clusters (Clusters 1–8), and the black line indicates the overall trend across all clusters.

A total of 649 lipid peaks were detected in positive and negative ion modes, including 509 peaks in positive ion mode and 140 peaks in negative ion mode, reflecting marked differences in ionisation behaviour among lipid species (Figure 2A). Analysis of lipid class composition showed that GLs represented the dominant lipid category, followed by GPs, whereas SPs, FAs, and PRs were present in much lower proportions. At the subclass level, TGs were the predominant components within GLs. GPs comprised several major subclasses together with a small number of minor subclasses, whereas SPs contained multiple molecular species, with Cer as the main subclass. Notably, CoQ was the only lipid detected within the PR category (Figure 2B).

To reveal the dynamic pattern of lipid accumulation during *C. camphora* seed kernel development, k-means clustering analysis was performed to classify all identified lipids into eight clusters (Clusters 1–8). Cluster 1 was enriched in GLs, GPs, SPs, and FAs, with GPs and GLs predominant, mainly including PCs (16:0/18:2) and PEs (18:1/18:3). This trend preliminarily suggests that membrane lipid anabolism may be relatively active at the early stage of seed development, and that the relative abundance of membrane lipids might gradually decline with kernel maturation. Cluster 5, the largest cluster, dropped sharply in abundance from S1 to S2 and then remained at a low level. This cluster mainly contained various GPs and SPs, excluding storage TGs, with typical representatives being LPC (16:0) and Cer (18:1/24:0). Its rapid decline in abundance at the early stage preliminarily implies that signalling lipids and membrane lipid precursors may be rapidly metabolised and utilised during initial seed development.

Cluster 7 exhibited a typical V-shaped pattern and was mainly composed of Cer, represented by Cer (18:1/16:0). Its initial decrease followed by a subsequent increase preliminarily indicates that such lipids may be involved in developmental regulation and stress response during the middle stage of seed development. The abundance of lipids in Cluster 8 decreased to the lowest level at S3 and then gradually increased. This cluster was mainly enriched in lipid components containing polyunsaturated fatty acids such as linoleic acid (18:2), MCFAs including decanoic acid (10:0) and lauric acid (12:0), as well as CoQ (CoQ10). This characteristic was highly consistent with the physiological maturation process and the late accumulation of functional lipids in *C. camphora* seed kernels.

In terms of developmental trends, Clusters 1 and 6 continuously declined throughout development; Clusters 2, 3, and 4 reached their maxima at S4, S2, and S3, respectively, before declining; Cluster 5, the largest cluster, decreased sharply from S1 to S2 and then remained at a low level; Cluster 7 showed a V-shaped pattern; and Cluster 8 decreased to its lowest level at S3 and then increased thereafter. These results indicate pronounced stage-dependent variation in lipid abundance during *C. camphora* seed kernel development (Figure 2C,D).

#### 2.2.2. Quantitative Analysis of Lipids in *C. camphora* Seed Kernels

**Figure 3:**
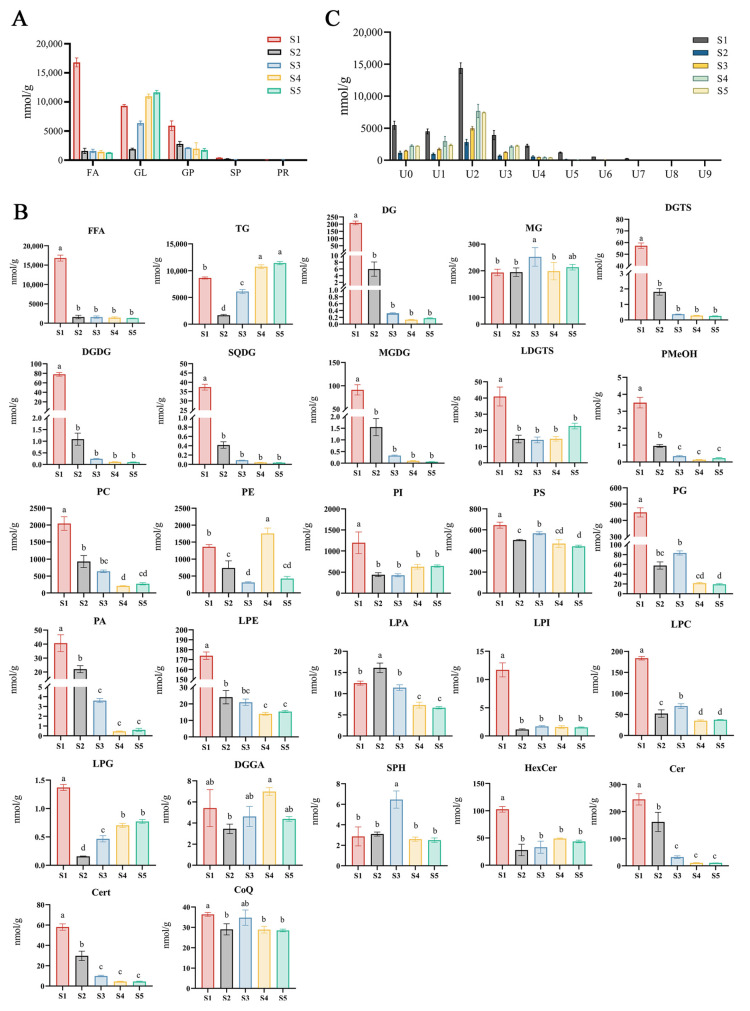
Quantitative analysis of the relative abundance of lipid molecules in *C. camphora* seed kernels at different developmental stages. (**A**,**B**) The abundances of all lipid compounds quantified within the same sample were summed to calculate the total relative abundances of major lipid categories and subclasses. Different letters indicate significant differences among developmental stages (*p* < 0.05). (**C**) Total unsaturated lipid content in *C. camphora* seeds.

Based on the quantitative lipidomic data, the relative peak intensities of lipid molecules in *C. camphora* seed kernels at stages S1–S5 were further analysed to characterise their developmental dynamics. At the class level, FA showed the highest relative abundance at S1, after which it decreased rapidly and remained low. In contrast, GL accumulated progressively from S1 to S5, despite a transient decrease between S1 and S2, and became the predominant lipid class at later developmental stages. GP showed an overall decreasing trend, reaching a minimum at the middle developmental stages before partially recovering during maturation. By comparison, SP and PR remained at consistently low levels throughout development and showed no obvious changes (Figure 3A).

A total of 27 major lipid subclasses were identified, and their accumulation patterns differed substantially during seed development. Within GLs, the major subclasses were TGs, DGs, and MGs. TGs decreased from S1 to S2 but then increased continuously from S2 to S5, closely matching the overall accumulation pattern of total GLs. DGs decreased from S1 to S2 and then remained at a very low and stable level, whereas MGs were relatively abundant at S3 and remained stable during the remaining stages. Within the GPs, the major subclasses displayed stage-specific changes. PEs, the most abundant GP subclass, declined markedly from S1 to S3 and then partially recovered at S4. PCs showed an overall decreasing trend from S1 to S5, with an early-stage pattern similar to that of PEs. PIs decreased from S1 to S2, increased from S2 to S4, and then stabilised at a relatively high level during the late stages. PSs, an important precursor for PE biosynthesis, remained stable throughout development. In addition, CoQ maintained a relatively high abundance from S1 to S5, whereas subclasses such as LPAs, LPGs, and PMeOH were detected only at low levels (Figure 3B).

To evaluate the overall degree of lipid unsaturation, unsaturated lipids in total seed lipids were quantified. Approximately 84.2% of the lipids in *C. camphora* seeds were unsaturated, and the number of double bonds ranged mainly from one to nine. Lipids containing one, two, or three double bonds accounted for the largest proportions, indicating that the lipid profile of *C. camphora* seed kernels is highly unsaturated (Figure 3C).

### 2.3. Quality Assessment of the Lipidomic Dataset

To assess the reliability of the lipidomic dataset and to characterise differences among developmental stages, multivariate statistical analyses were performed. Principal component analysis (PCA) showed that the first two principal components, PC1 and PC2, explained 74.71% and 13.48% of the total variance, respectively. Biological replicates within each stage clustered closely together, whereas samples from different developmental stages were clearly separated, indicating good reproducibility and marked stage-dependent differences in lipid profiles (Figure S2A).

In addition, the coefficient of variation (CV) values of lipid peaks were highly concentrated in the quality control (QC) samples, with more than 75% of lipid features showing CV values below 0.3, indicating good analytical stability and satisfactory technical reproducibility (Figure S2B).

To identify significantly altered lipids among developmental stages, pairwise comparisons were conducted using the criteria VIP > 1, FC ≥ 2 or FC ≤ 0.5, and *p* < 0.05. Venn diagram analysis showed that differentially abundant lipids exhibited distinct distribution patterns among comparisons, with some lipids shared across all comparison groups (Figure S2C). Collectively, these results demonstrated substantial dynamic remodelling of the lipidome during *C. camphora* seed kernel development.

### 2.4. Differentially Abundant Lipids in C. camphora Seeds at Different Developmental Stages

**Figure 4:**
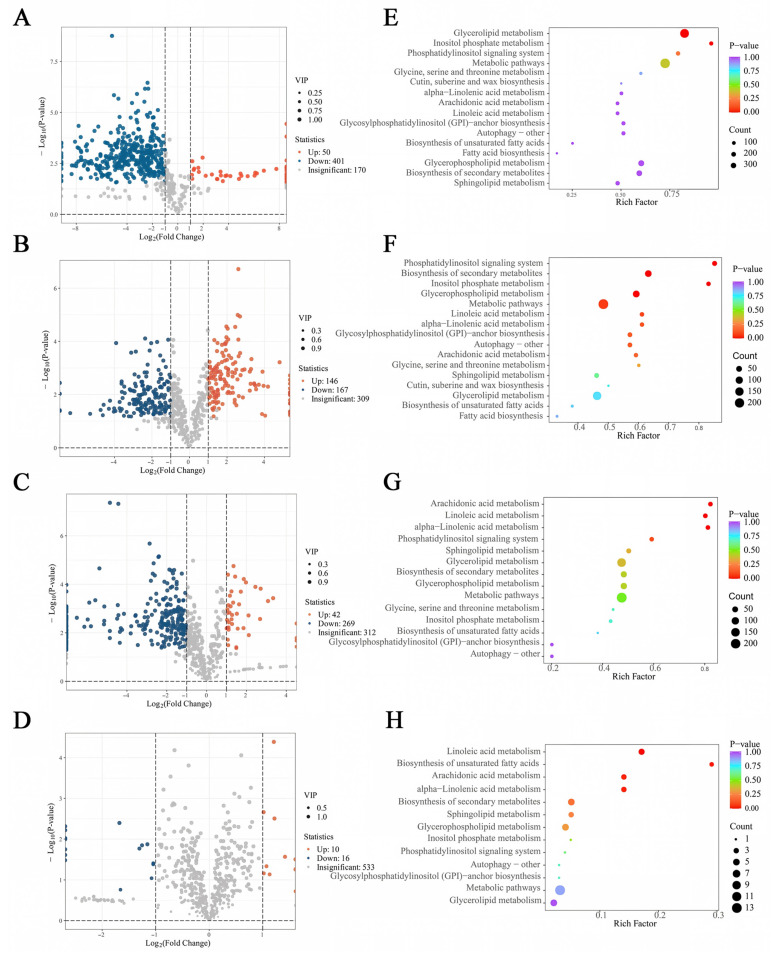
Differentially abundant lipids in *C. camphora* seed kernels at different developmental stages. (**A**–**D**) Volcano plots showing differentially abundant lipids in the comparisons A/E S2 vs. S1, B/F S3 vs. S2, C/G S4 vs. S3, and D/H S5 vs. S4, respectively. Lipids with VIP > 1, fold change ≥ 2 or ≤0.5, and *p* < 0.05 were defined as significantly differentially abundant. (**E**–**H**) KEGG pathway enrichment analysis of differentially abundant lipids in the four comparison groups.

Volcano plot analysis showed that the S1 vs. S2 comparison contained the largest number of differentially abundant lipids, with 451 lipids identified, including 50 upregulated and 401 downregulated molecules (Figure 4A). In the S2 vs. S3 comparison, 313 differential lipids were detected, comprising 146 upregulated and 167 downregulated lipids (Figure 4B). The S3 vs. S4 comparison yielded 311 differential lipids, including 42 upregulated and 269 downregulated species (Figure 4C). By contrast, the S4 vs. S5 comparison showed the fewest changes, with only 26 differential lipids, including 10 upregulated and 16 downregulated molecules (Figure 4D). These results indicated that the most extensive lipidomic remodelling occurred during the early developmental transition from S1 to S2.

The KEGG pathway enrichment analysis was then performed for differential lipids identified in the four consecutive stage comparisons. In S1 vs. S2, GL metabolism and inositol phosphate metabolism were the most significantly enriched pathways, with 266 differential lipids enriched in glycerolipid metabolism and 22 in inositol phosphate metabolism. In S2 vs. S3, the significantly enriched pathways included GP metabolism, metabolic pathways, and biosynthesis of secondary metabolites. In S3 vs. S4, differential lipids were mainly enriched in arachidonic acid metabolism, linoleic acid metabolism, and α-linolenic acid metabolism, while GL metabolism and GP metabolism also remained highly enriched. In S4 vs. S5, linoleic acid metabolism, biosynthesis of unsaturated fatty acids, and arachidonic acid metabolism showed the strongest enrichment, whereas the enrichment of GL metabolism was relatively reduced (Figure 4E–H).

### 2.5. Dynamic Changes and Fatty Acid Composition of TGs, DGs, PEs, and PCs

Based on the studies, GLs and GPs were the two dominant lipid categories in *C. camphora* seed kernels, with four representative subclasses: TGs and DGs from GLs, and PEs and PCs from GPs, which were selected for further analysis. The numbers and proportions of significantly upregulated and downregulated lipid molecules were compared across developmental stages. Lipid molecules of TGs, DGs, PEs and PCs exhibited distinct dynamic changes during seed development.

**Figure 5:**
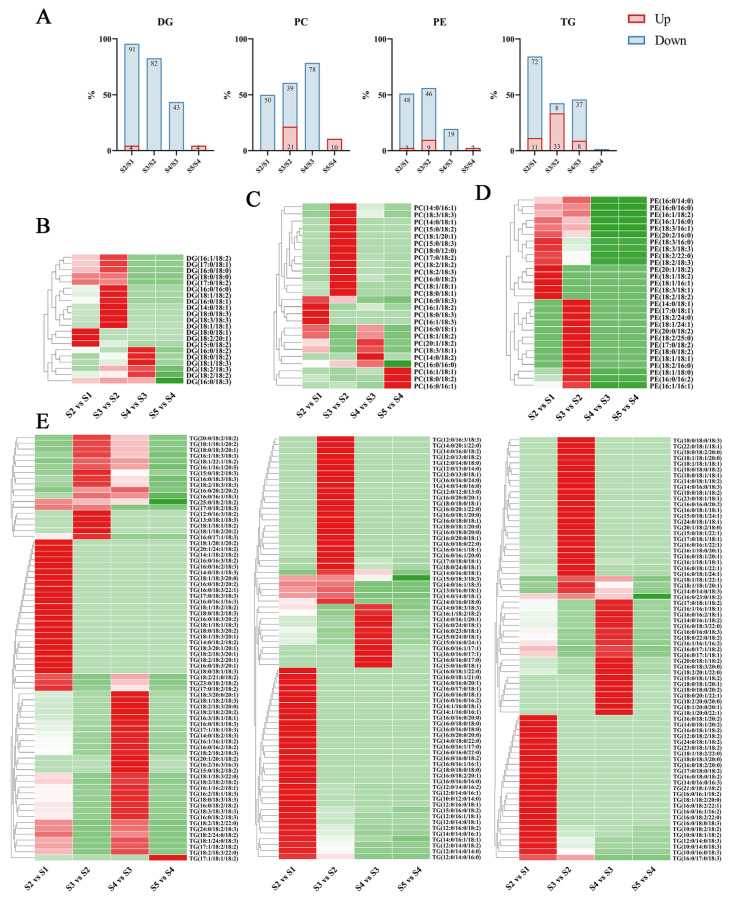
Differential changes in TG, DG, PE, and PC lipid molecules in *C. camphora* seed kernels across developmental stages. (**A**) Numbers of upregulated and downregulated TG, DG, PE, and PC lipid molecules. Blue bars indicate downregulation, and red bars indicate upregulation; the exact numbers are labelled above the bars. (**B**) Differential DG lipid molecules and their fold changes. (**C**) Differential PC lipid molecules and their fold changes. (**D**) Differential PE lipid molecules and their fold changes. (**E**) Differential TG lipid molecules and their fold changes. In the heatmaps, red and green indicate upregulation and downregulation, respectively. Differential lipid molecules were screened according to the criteria of VIP > 1, FC ≥ 2 or FC ≤ 0.5, and *p* < 0.05.

DGs were predominantly downregulated in S2/S1 and S3/S2, accounting for 91% and 82% of total DG molecules, respectively, and the proportion of downregulated DGs gradually decreased towards seed maturation. PCs were mainly downregulated in S2/S1 and S4/S3, but were predominantly upregulated in S3/S2, after which they remained relatively stable. PEs were chiefly downregulated during the early developmental stages, with only a few upregulated molecules, and their abundance decreased markedly at later stages. In contrast, TGs were strongly upregulated in S2/S1 and S3/S2; in S2/S1, 72% of TG molecules were upregulated, whereas 11% were downregulated. This trend shifted towards downregulation in S4/S3, consistent with the overall developmental pattern of total TG abundance (Figure 5A–E).

**Figure 6:**
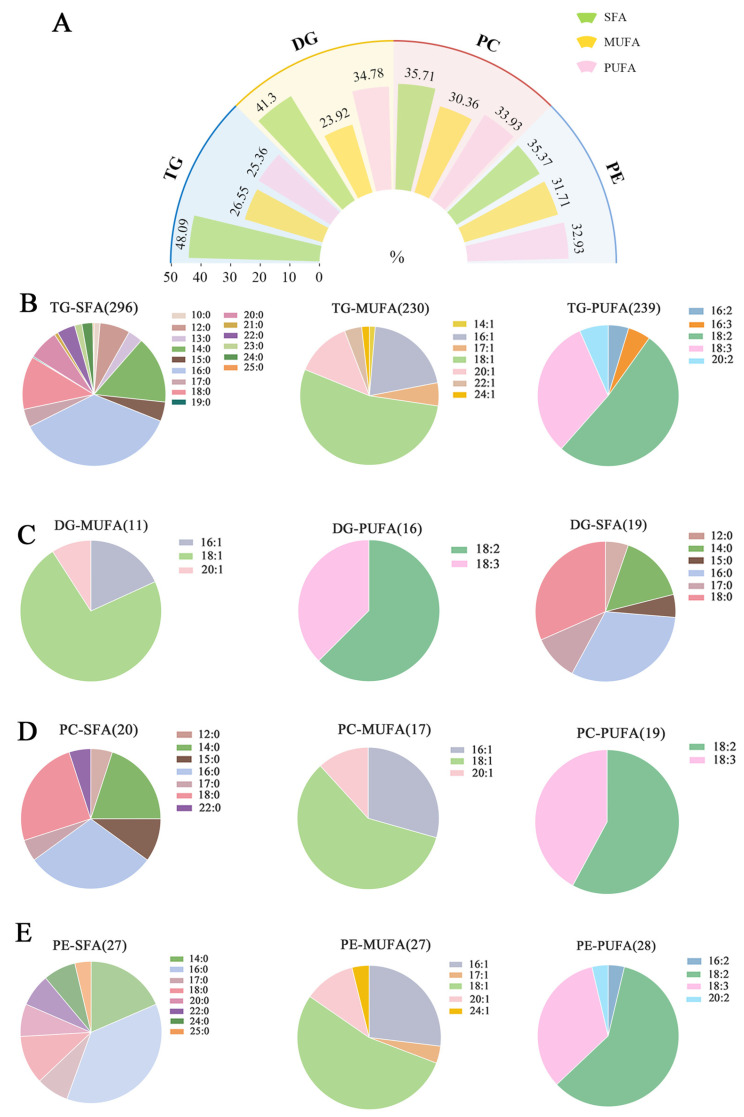
Fatty acid composition of TG, DG, PC, and PE lipid molecules in *C. camphora* seed kernels. (**A**) Proportions of saturated fatty acids (SFAs), monounsaturated fatty acids (MUFAs), and polyunsaturated fatty acids (PUFAs) in TG, DG, PC, and PE lipid molecules. (**B**–**E**) Fatty acid profiles of TG, DG, PC, and PE lipid molecules, respectively, together with the numbers of SFAs, MUFAs, and PUFAs. Different coloured sectors in the pie charts represent the proportions of each fatty acid type, and the numbers in parentheses indicate the corresponding numbers of fatty acids.

Lipids consist mostly of a glycerol backbone esterified to fatty acids, so the fatty acid compositions of TGs, DGs, PCs, and PEs were further examined [33]. In TGs, the fatty acid moieties were mainly composed of saturated fatty acids (SFAs), monounsaturated fatty acids (MUFAs), and polyunsaturated fatty acids (PUFAs), accounting for 44.71%, 27.38%, and 28.45%, respectively (Figure 6A). Overall, unsaturated fatty acids accounted for 65.83% of the total fatty acid composition in TGs. Among the SFAs, palmitic acid (16:0, 36.12%) was the predominant component, together with medium-chain fatty acids such as decanoic acid (10:0), lauric acid (12:0), and myristic acid (14:0). Within the MUFAs, oleic acid (18:1, 52.17%) was the major component, whereas linoleic acid (18:2, 46.03%) and linolenic acid (18:3, 28.45%) were the principal PUFAs (Figure 6B).

In DGs, SFAs, MUFAs, and PUFAs accounted for 41.30%, 23.91%, and 34.78%, respectively (Figure 6A), and unsaturated fatty acids comprised 58.69% of the total fatty acid composition. The major representative fatty acids were palmitic acid (16:0, 31.58%), oleic acid (18:1, 72.73%), linoleic acid (18:2, 62.50%), and linolenic acid (18:3, 37.50%) (Figure 6C).

In PCs, the proportions of SFAs, MUFAs, and PUFAs were relatively similar, at 35.71%, 30.36%, and 33.93%, respectively, whereas unsaturated fatty acids were 64.29% of the total. The predominant fatty acids were palmitic acid (16:0, 30.00%), oleic acid (18:1, 58.82%), linoleic acid (18:2, 57.89%), and linolenic acid (18:3, 42.11%) (Figure 6D).

Similarly, in PEs, the proportions of SFAs, MUFAs, and PUFAs were 32.93%, 32.93%, and 34.15%, respectively, and unsaturated fatty acids accounted for 67.08% of the total fatty acid composition. The major representative fatty acids were palmitic acid (16:0, 37.04%), oleic acid (18:1, 51.85%), linoleic acid (18:2, 57.14%), and linolenic acid (18:3, 32.14%) (Figure 6E). Taken together, these results indicate that the major lipid subclasses in *C. camphora* seed kernels are characterised by a high degree of unsaturation.

## 3. Discussion

Cross-sectional observations showed that embryo development in *C. camphora* seeds lagged markedly behind fruit morphogenesis and seed coat hardening. This pattern differs from that reported in other fatty oil seed species, such as *Carya illinoinensis* and *Camellia oleifera*. In *C. illinoinensis*, embryo tissues develop asynchronously and exhibit spatial heterogeneity in composition [34], whereas in *C. oleifera*, embryo growth is more closely coordinated with fruit expansion and dry matter accumulation [35]. In contrast, in *C. camphora*, fruit enlargement, seed coat differentiation, and endocarp lignification were largely completed by 81 DAF, whereas embryo differentiation and cotyledon formation were not evident until 81–102 DAF. Another notable feature was the prolonged presence of a transparent viscous fluid within the seed cavity before embryo formation; this fluid gradually became gel-like as the cotyledons developed and eventually filled the cavity. Taken together, these observations indicate a distinctive developmental programme in which morphological maturation precedes physiological maturity. Consistent with this pattern, kernel size and oil content continued to increase even after the external fruit morphology had stabilised, and seed maturation, dehydration, and reserve accumulation were completed by mid-October. This period is therefore likely to represent the optimal harvest window for *C. camphora* seeds.

Oilseeds are important dietary sources of lipids, which are essential macronutrients for human health [36]. Comprehensive lipidomic analysis identified 627 lipid molecules in *C. camphora* seed kernels, spanning 27 subclasses within five major categories, including 377 GLs, 169 GPs, and 56 SPs. Compared with several common oilseed crops, *C. camphora* seed kernels exhibited relatively high lipid diversity [26,27,37]. As in many other oilseed species, GLs and GPs were the dominant lipid categories. Notably, the GP fraction was relatively abundant and diverse. Because GPs serve as important functional lipids and natural emulsifiers in the food, pharmaceutical, and personal care industries, *C. camphora* seed kernels may represent a promising alternative source of GP-rich raw materials [38].

Lipid chain length and degree of unsaturation are important structural characteristics that largely determine the physicochemical properties of lipids, including membrane fluidity, melting point, oxidative stability and digestibility [39]. In this study, approximately 84.2% of the total lipids in *C. camphora* seed kernels were unsaturated, with double bond numbers mainly ranging from one to nine. Lipids containing one to three double bonds were predominant, indicating that *C. camphora* seed kernels exhibit an extremely high unsaturation level, and all major lipid subclasses share this highly unsaturated characteristic. From the perspective of plant physiology, unsaturated lipids have low melting points, which can maintain cell membrane fluidity and structural integrity during seed maturation, desiccation and ambient temperature fluctuations [40]. It is therefore speculated that the abundant accumulation of unsaturated lipids in *C. camphora* seeds may serve as a physiological regulation strategy to adapt to seed development and external environmental changes.

Fatty acid composition and relative abundance are key determinants of oil quality. According to fatty acid chain length, TGs can be classified as short-chain triglycerides (SCTs, C < 8), medium-chain triglycerides (MCTs, 8 ≤ C ≤ 12), and long-chain triglycerides (LCTs, C > 12) [38]. Most conventional fatty oil seed crops, including *Camellia oleifera* [38], *Juglans regia* [41,42], and *Olea europaea* [43], are dominated by LCFAs. In contrast, *C. camphora* seed kernel oil is enriched in MCFAss, particularly decanoic acid and lauric acid, indicating a distinctive medium-chain lipid profile. MCTs have been associated with rapid energy supply and beneficial effects on body weight and glucose metabolism [44]. Compared with coconut oil, another typical medium-chain oil, *C. camphora* seed kernel oil remains liquid at room temperature and appears to possess a more balanced fatty acid composition [45]. Lipidomic analysis further showed that glycerolipids accounted for 60.13% of total lipids and that TGs were the predominant subclass (44.66%), consistent with the role of GLs as the principal storage lipids in oilseeds [46,47]. These results highlight the potential of *C. camphora* seed kernels as a novel functional fatty oil resource. Another notable finding was the dynamic accumulation of CoQ, which increased rapidly during early development, declined gradually during the middle and late stages, and then remained relatively stable. This pattern is consistent with the proposed roles of CoQ in energy metabolism, oxidative protection, and the maintenance of storage lipid stability during seed development [48]. Recent studies have demonstrated that CoQ serves as an electron carrier in the mitochondrial respiratory chain to drive ATP synthesis, satisfying the high energy requirements during early seed development. Meanwhile, CoQ acts as a lipophilic antioxidant to protect cell membranes and nascent storage lipids from oxidative damage [49]. Although CoQ content gradually decreases at the middle and late developmental stages, its sustained basal level can alleviate oxidative stress during seed maturation, thereby maintaining seed viability and oil quality. Nuts and fatty oil seed crops are recognised as important dietary sources of CoQ [50]. The CoQ content in *C. camphora* seed kernels appears to be comparable to that reported for pistachios and peanuts, but higher than that in traditional oilseed crops such as legumes and rapeseed [51,52,53], suggesting that *C. camphora* seeds may represent a sustainable natural source of CoQ. In addition, *C. camphora* seeds are rich in MCFAs, particularly decanoic and lauric acids, and the coexistence of these fatty acids with relatively high CoQ levels may contribute to lipid antioxidant capacity and seed longevity [54].

The Kennedy pathway (Figure S3) illustrates lipid metabolic rewiring during *C. camphora* seed kernel development. As the core acyl donor, acyl-CoA sequentially feeds into LPA, PA, and DG biosynthesis. As a key metabolic hub, DGs are partitioned between storage lipid TGs and membrane phospholipid PCs/PEs. Developmental changes in these lipids reveal a temporal flux shift: early stages prioritise PCs/PEs for membrane biogenesis, while middle/late stages favour TG accumulation. TGs are enriched in MCFAs (C10:0, C12:0), whereas PCs/PEs contain more long-chain/unsaturated species (C16:0, C18:1, C18:2). These differences likely reflect enzyme substrate preferences, aligning with stage-specific lipid accumulation patterns. The Kennedy pathway thus coordinates membrane biogenesis and storage lipid synthesis to support seed development [55]. DGs, as neutral lipids, are both a central intermediate in lipid metabolism and a precursor of GP biosynthesis [56]. The high abundance of DGs at S1 may reflect active lipid biosynthesis during early seed development, whereas their sharp decline and persistently low levels from S2 to S5 suggest rapid conversion into storage lipids such as TGs during maturation. These findings indicate that DGs may play a transitional role in lipid interconversion during seed development. Further multi-omics studies will be required to elucidate the regulatory mechanisms underlying the synthesis and turnover of these abundant lipid species.

The dynamic lipid changes in *C. camphora* seed kernels are closely related to oil quality, nutrition and lipid accumulation mechanisms. MCFAs (C10:0, C12:0) gradually increased from S3 and peaked at S5, accounting for 10–15% of total lipids, which coincided with seed maturation and contributed to the formation of MCT-rich oil. Free fatty acids (FFAs) remained at a low level of 1–2% after S2, which is beneficial to oil extraction. Meanwhile, GPs, including PEs, LPCs and LPEs, were maintained at extremely low levels at S5, avoiding adverse effects on oil flavour and reflecting the high edible quality of *C. camphora* seed oil [57]. MCTs also endow the oil with unique physiological functions related to energy supply and metabolic regulation [58].

The decrease in DGs, PCs and PEs from S1 to S2 indicates a metabolic transition from membrane lipid synthesis to storage lipid accumulation, which is supported by KEGG enrichment in GL and GP pathways. From S2 to S5, continuous TG accumulation accompanied by low PC and PE levels suggests that DGs act as a key intermediate converted into TGs via the DGAT pathway, while PCs and PEs serve as acyl donors involved in lipid remodelling [59]. The distinct fatty acid composition among lipid subclasses (C10:0/C12:0 dominated in TGs, whereas C16:0/C18:1/C18:2 were enriched in PCs/PEs) is attributed to the substrate selectivity of Kennedy pathway enzymes, in agreement with the distinct substrate preferences of DGAT and PDAT [58]. In conclusion, lipid dynamics are closely associated with TG biosynthesis, fatty acid remodelling and oil quality formation. Comprehensively considering the highest MCT accumulation, low FFA content and minimal phospholipid abundance at S5, it is speculated that S5 may serve as the suitable harvest window to potentially maximise MCT enrichment and maintain the optimal flavour and nutritional properties of *C. camphora* seed kernel oil. Several limitations should be acknowledged in this study. First, although we observed a decline in MGDG and DGDG levels during seed maturation, our current data do not allow us to trace the metabolic fate of their fatty acyl moieties, so it remains unclear whether these moieties are directed toward catabolism or are recycled into other polar lipids or TGs via DGs or acyl editing pathways. Second, although we divided the developmental stages based on morphological changes over a total period of 183 days, the sampling intervals were long and uneven (e.g., S2 lasts 49 days and S4 lasts 69 days). With only five time points, it is impossible to fit a standard sigmoidal kinetic curve, and it is difficult to distinguish between lipid biosynthesis, degradation, and interconversion. Therefore, we cannot calculate the net synthesis or degradation rates of TGs and other lipids, nor can we correlate lipid accumulation with individual fruit mass gain. Third, this study only focused on the dynamic changes in lipid profiles during seed development, without investigating the changes in lipid composition and content during seed storage, which limits our understanding of oil quality stability during post-harvest storage.

These limitations highlight important directions for future research. First, given that storage conditions (e.g., temperature, humidity, time) can affect oil quality through lipid oxidation or hydrolysis, subsequent studies should systematically analyse the stability of *C. camphora* seed kernel oil during post-harvest storage, clarify the dynamic patterns of lipid changes during storage, and thereby reveal the mechanisms underlying oil quality alteration, providing a basis for proper seed preservation and commercial application. Meanwhile, to elucidate the precise metabolic fate of GL-derived fatty acids during seed maturation, integrated approaches such as metabolic flux analysis or enzyme activity assays should be employed. Furthermore, future research should adopt denser time-course sampling (e.g., every 7–10 days) focusing on the rapid accumulation and maturation stages, combined with metabolic tracing techniques to conduct rigorous kinetic analyses. At the same time, dynamic monitoring of single-seed biomass (fresh/dry weight) at each developmental stage, together with absolute lipid quantification on a per-seed basis, will enable calculation of TG synthesis and degradation rates, thereby providing deeper insights into the lipid remodelling patterns during *C. camphora* seed development.

## 4. Materials and Methods

### 4.1. Plant Materials

*C. camphora* seeds were collected from three healthy and uniformly grown mother trees, which were screened from an approximately 15-hectare homogeneous clonal plantation in Lehua Town, Nanchang, Jiangxi Province, China. All individuals in this plantation belong to the same clonal line with identical genetic background, and the site conditions, soil properties, and microenvironments across the plantation are highly consistent. Sampling was conducted from late April to late October 2025, covering a total seed developmental period of 183 days. At each developmental stage, 30 plump seeds were collected from the four cardinal directions of each mother tree and pooled as one composite sample, with three technical replicates prepared for subsequent analysis.

For paraffin section preparation, freshly collected intact seeds were directly used. For lipidomic analysis, the exocarp and seed coat were carefully removed, and the isolated seed kernels were immediately flash-frozen in liquid nitrogen. The frozen samples were then transported to the laboratory, immediately freeze-dried to remove most of the free water, and subsequently stored at −80 °C until further analysis. This freeze-drying step substantially inhibits the activity of lipolytic enzymes (e.g., phospholipase D) that could otherwise remain active even at −80 °C, thereby minimising the risk of artificial artifacts such as diacylglycerols and lyso-lipids.

### 4.2. Paraffin Sections

Paraffin sectioning was performed to examine the morphological and anatomical changes in *C. camphora* seeds during development. Samples were collected every 10 days from late April to late October 2025, with 15 seeds harvested at each time point. Some seeds were cut longitudinally for observation under a stereomicroscope (Olympus Corporation, Tokyo, Japan). The remaining seeds were cleaned and fixed in 70% formalin–acetic acid–alcohol (FAA) solution (all chemicals were purchased from Sinopharm Chemical Reagent Co., Ltd., Shanghai, China) for 48 h. The samples were then dehydrated with a graded ethanol series (Sinopharm Chemical Reagent Co., Ltd., Shanghai, China), cleared in xylene (Sinopharm Chemical Reagent Co., Ltd., Shanghai, China), and embedded in soft paraffin (Leica Biosystems, Nussloch, Germany) before sectioning. The sections were stained with safranin (Sigma-Aldrich, St. Louis, MO, USA) and fast green (Sigma-Aldrich, St. Louis, MO, USA), mounted with neutral balsam (Sinopharm Chemical Reagent Co., Ltd., Shanghai, China), dried at 37 °C for 12 h, and observed under a Leica light microscope (Leica Microsystems, Wetzlar, Germany) [60].

### 4.3. Chemical Reagents

Chromatography-grade acetonitrile (ACN), methanol (MeOH), isopropanol (IPA), dichloromethane (CH_2_Cl_2_) and methyl tert-butyl ether (MTBE) were commercially available from Merck (Darmstadt, Germany). Chromatography-grade formic acid (FA) and ammonium formate (AmFA) were sourced from Sigma-Aldrich (St. Louis, MO, USA). A Milli-Q system (Millipore, Billerica, MA, USA) was used to produce ultrapure water for the experiment. Lipid reference standards were supplied by Sigma-Aldrich and Avanti Polar Lipids (Alabaster, AL, USA). Additional experimental instruments included a 5424R high-speed refrigerated centrifuge (Eppendorf, Hamburg, Germany), AS 60/220.R2 electronic balance (RADWAG, Radom, Poland), MM400 ball mill (Retsch, Haan, Germany), CentriVap vacuum concentrator (LABCONCO, Kansas City, MO, USA), MIX-200 multi-tube vortex oscillator (Jingxin, Kunshan, China), and KQ5200E ultrasonic cleaner (Shumei, Kunshan, China).

### 4.4. Sample Preparation and Lipid Extraction

Lipid extraction was performed with a mixed solvent of methanol and methyl tert-butyl ether (MTBE) according to the method described by Matyash et al. [61], with minor modifications. After freeze-drying, 20 mg of powdered sample was weighed into a centrifuge tube containing steel beads. Then, 1 mL of MTBE/MeOH (3:1, *v*/*v*) extraction solvent was supplemented, and the mixture was subjected to vortexing for 30 min. Next, 300 μL of ultrapure water was added, and the mixture was vortexed for 1 min. After standing at 4 °C for 10 min, the mixture was centrifuged at 12,000 rpm for 3 min. An aliquot of 400 μL supernatant was collected and concentrated to complete dryness at 20 °C. The residue was reconstituted with 200 μL of ACN/IPA (1:1, *v*/*v*), accompanied by 3 min of vigorous vortexing. After centrifugation again at 12,000 rpm and 4 °C for 3 min, 120 μL of the final supernatant was collected for LC-MS/MS analysis.

### 4.5. Lipidomics Analysis

#### 4.5.1. HPLC Conditions

Sample extracts were analysed by LC-ESI-MS/MS using an ExionLC AD UPLC system (SCIEX, Framingham, MA, USA) coupled with a QTRAP^®^ 6500+ mass spectrometer (SCIEX, Framingham, MA, USA). Chromatographic separation was performed on a Thermo Accucore^™^ C30 column (2.6 μm, 2.1 mm × 100 mm i.d., Thermo Fisher Scientific, Waltham, MA, USA). The mobile phase consisted of solvent A (acetonitrile/water, 60:40, *v*/*v*, containing 0.1% formic acid and 10 mmol/L ammonium formate) and solvent B (acetonitrile/isopropanol, 10:90, *v*/*v*, containing 0.1% formic acid and 10 mmol/L ammonium formate). The gradient elution was set as follows: 0 min, 80:20 (A/B, *v*/*v*); 2.0 min, 70:30; 4.0 min, 40:60; 9.0 min, 15:85; 14.0 min, 10:90; 15.5–17.3 min, 5:95; 17.3–20.0 min, returning to 80:20. The flow rate was 0.35 mL/min, column temperature was maintained at 45 °C, and the injection volume was 2 μL. The eluate was subsequently introduced into an ESI-triple quadrupole–linear ion trap (QTRAP) mass spectrometer for detection. The LC-MS/MS analytical procedure followed the methodology by Xuan et al. [62], with appropriate modification.

#### 4.5.2. ESI-MS/MS Conditions

A Sciex QTRAP^®^ 6500+ LC-MS/MS triple quadrupole–linear ion trap mass spectrometer (SCIEX, Framingham, MA, USA) fitted with a Turbo Ion-Spray electrospray interface was employed. LIT and QQQ scans were performed in both positive and negative ion modes, with instrument operation controlled by Analyst 1.6.3 software (SCIEX, Framingham, MA, USA). The ion source and gas parameters were configured, and instrument tuning as well as mass calibration were completed using polypropylene glycol solutions (Sigma-Aldrich, St. Louis, MO, USA). Data acquisition was carried out in MRM mode, mass spectral parameters for each ion transition were optimised, and target components were qualitatively and quantitatively monitored based on the retention times of the metabolites.

Lipid annotation and identification were performed based on the in-house MetWare lipid database (MWDB) according to the method described by Eriksson et al. [63], with minor modifications. Lipid species were qualitatively identified by matching characteristic MRM precursor/product ion pairs and retention time information. The QTRAP^®^ 6500+ platform operated in multiple reaction monitoring (MRM) mode was used for targeted lipidomics analysis, which eliminated interference ions and ensured accurate identification and quantification. Quality control (QC) samples were inserted periodically throughout the LC-MS/MS run to evaluate system stability and data reproducibility. The retention time and peak intensity consistency of total ion current (TIC) were assessed, and Pearson correlation analysis of QC samples was performed according to Eriksson et al. [63]. Only lipids with acceptable coefficient of variation (CV) in QC samples were retained for subsequent differential analysis. Lipid quantification was calculated by the internal standard method based on chromatographic peak area integration.

### 4.6. Data Analysis

The data were preliminarily collated with Excel 2019 (Microsoft, Redmond, WA, USA). One-way ANOVA was conducted with SPSS 27.0 (IBM, Armonk, NY, USA), and Duncan’s multiple range test was adopted for multiple comparisons at *p* < 0.05. Figures were constructed with GraphPad Prism 9.0 (GraphPad Software, San Diego, CA, USA) and Origin 2024 (OriginLab, Northampton, MA, USA) and were further refined in Adobe Illustrator 2022 (Adobe, San Jose, CA, USA). All experiments were carried out in three independent replicates, and experimental data were expressed as the mean ± standard deviation (SD).

The fatty acid composition of each lipid class (TGs, DGs, PCs, PEs) was determined as follows. All detected lipid molecules within a given class were considered. For each such lipid molecule, the constituent fatty acyl chains were identified based on MS/MS fragmentation patterns. Each fatty acyl chain was classified as SFAs, MUFAs, or PUFAs. The percentage of a given fatty acid (e.g., 16:0) or a given type (e.g., SFAs) was calculated as the number of chains of that type divided by the total number of fatty acyl chains from all lipid molecules of that class, multiplied by 100%. This approach reflects the relative abundance of fatty acyl chains at the molecular species level, not the proportion of lipid molecules containing such chains, nor the positional distribution within individual molecules.

## 5. Conclusions

In this study, LC-MS/MS was used to systematically characterise the lipidomic features and dynamic accumulation patterns of *C. camphora* seed kernels across five developmental stages. The results showed that seed development followed a distinctive pattern in which morphological maturation preceded physiological maturity. A total of 627 lipid molecules were identified, and the lipid profile was characterised by a high degree of unsaturation. GLs were the predominant lipid category, with TGs serving as the principal storage lipids. The fatty acid composition was enriched in MCFAs, especially decanoic acid and lauric acid. In addition, the seed kernels accumulated relatively abundant and stable CoQ, highlighting their potential nutritional and antioxidant value. Differentially abundant lipids were concentrated mainly in the early developmental stages and were significantly enriched in GL and GP metabolic pathways. DGs, PCs, and PEs decreased during early development, whereas TGs accumulated continuously from the middle stage onwards. Specifically, MCFAs gradually increased from S3 to a peak at S5, while CoQ showed an early increase followed by a gradual decrease, consistent with its roles in energy supply and antioxidant protection. Overall, this study clarifies the lipid accumulation pattern and metabolic characteristics of *C. camphora* seed kernels and provides a basis for their future development as a novel functional fatty oil resource.

## Data Availability

The raw data supporting the conclusions of this article will be made available by the authors on request.

## Supplementary Materials

The following supporting information can be downloaded at: https://www.mdpi.com/article/10.3390/plants15121887/s1. Table S1. Classification of lipids identified in *C*. *camphora* seeds. Figure S1. Overlapped total ion chromatograms (TICs) of QC samples in negative and positive ionisation modes. (A) Negative ionisation mode; (B) positive ionisation mode. The high overlap of retention time and peak intensity across all QC samples indicates good stability and repeatability of the LC-MS system during lipidomic analysis. Figure S2. Overview analysis of lipidomics in *C*. *camphora* seed kernels. (A) Principal component analysis of QC samples. (B) CV distribution of lipid molecules. (C) Venn diagram of differential lipid molecules. Figure S3. The Kennedy pathway of lipid metabolism in *C. camphora* seed kernels. The diagram illustrates the metabolic connections of key lipid intermediates; the dynamic changes in DG, PC, PE and TG contents across five developmental stages; and the distinct fatty acid composition characteristics of different lipid components involved in this pathway.

## Abbreviations

The following abbreviations are used in this manuscript:

Cer	Ceramide
CoQ	Coenzyme Q
CV	Coefficient of variation
DAF	Days after flowering
DG	Diacylglycerol
DGAT	Diacylglycerol acyltransferase
ESI	Electrospray ionisation
FA	Fatty acid/Fatty acyl
FAA	Formalin–acetic acid–alcohol
FC	Fold change
GL	Glycerolipid
GP	Glycerophospholipid
HPLC	High-performance liquid chromatography
KEGG	Kyoto Encyclopedia of Genes and Genomes
LC-MS/MS	Liquid chromatography–tandem mass spectrometry
LCT	Long-chain triglyceride
LPA	Lysophosphatidic acid
LPC	Lysophosphatidylcholine
LPG	Lysophosphatidylglycerol
MCT	Medium-chain triglyceride
MeOH	Methanol
MG	Monoacylglycerol
MRM	Multiple reaction monitoring
MTBE	Methyl tert-butyl ether
MUFA	Monounsaturated fatty acid
PA	Phosphatidic acid
PC	Phosphatidylcholine
PCA	Principal component analysis
PE	Phosphatidylethanolamine
PG	Phosphatidylglycerol
PI	Phosphatidylinositol
PMeOH	Phosphatidylmethanol
PUFA	Polyunsaturated fatty acid
QC	Quality control
SD	Standard deviation
SFA	Saturated fatty acid
SP	Sphingolipid
TG	Triacylglycerol
VIP	Variable importance in the projection
