# Supplementary material for: Quantitative Lipidomics Reveals Dynamic Lipid Profiles in Cinnamomum camphora Seed Kernels at Different Developmental Stages
Source: Plants (Basel). 2026 Jun 17;15(12):1887. doi: 10.3390/plants15121887 (PMC13306347; doi:10.3390/plants15121887)
Supplement: Supplementary file 1 [file plants-15-01887-s001.zip › plants-4335750-supplementary.pdf]

Category	Sub class	Number
Glycerolipids, GLs	Diglyceride, DG	23
Glycerolipids, GLs	Digalactosyldiacylglycerol, DGDG	15
Glycerolipids, GLs	Diacylglycerylglucuronide, DGGA	1
Glycerolipids, GLs	Diacylglyceryltrimethylhomoserine, DGTS	20
Glycerolipids, GLs	Lyso-diacylglyceryltrimethylhomoserine, LDGTS	5
Glycerolipids, GLs	Monoglyceride, MG	3
Glycerolipids, GLs	Monogalactosyldiacylglycerol, MGDG	16
Glycerolipids, GLs	Sulfoquinovosyldiacylglycerol, SQDG	14
Glycerolipids, GLs	Triglyceride, TG	280
Glycerophospholipids, GPs	Lysophosphatidic acid, LPA	8
Glycerophospholipids, GPs	Lysophosphatidylcholine, LPC	15
Glycerophospholipids, GPs	Lysophosphatidylethanolamine, LPE	8
Glycerophospholipids, GPs	Lysophosphatidylglycerol, LPG	2
Glycerophospholipids, GPs	Lysophosphatidylinositol, LPI	3
Glycerophospholipids, GPs	Phosphatidic acid, PA	11
Glycerophospholipids, GPs	Phosphatidylcholine, PC	28
Glycerophospholipids, GPs	Phosphatidylethanolamine, PE	41
Glycerophospholipids, GPs	Phosphatidylglycerol, PG	20
Glycerophospholipids, GPs	Phosphatidylinositol, PI	19
Glycerophospholipids, GPs	Phosphatidylmethanol, PMeOH	4
Glycerophospholipids, GPs	Phosphatidylserine, PS	10
Sphingolipids, SPs	Ceramides, Cer	14
Sphingolipids, SPs	Ceramide transfer protein, Cert	20
Sphingolipids, SPs	Hexosylceramide, Hexcer	16
Sphingolipids, SPs	Sphingosine, SPH	6
PrenolLipids, PR	Coenzyme Q, CoQ	3
Fatty Acyls	Free Fatty Acids, FAA	22

Table S1. Classification of lipids identified in *C. camphora* seeds.

A

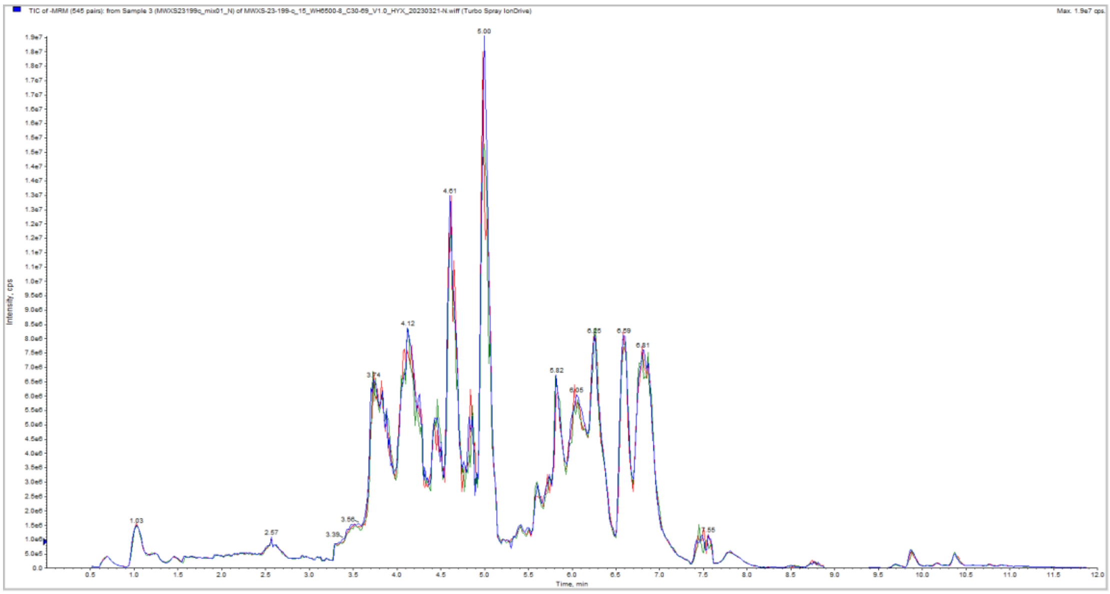

B

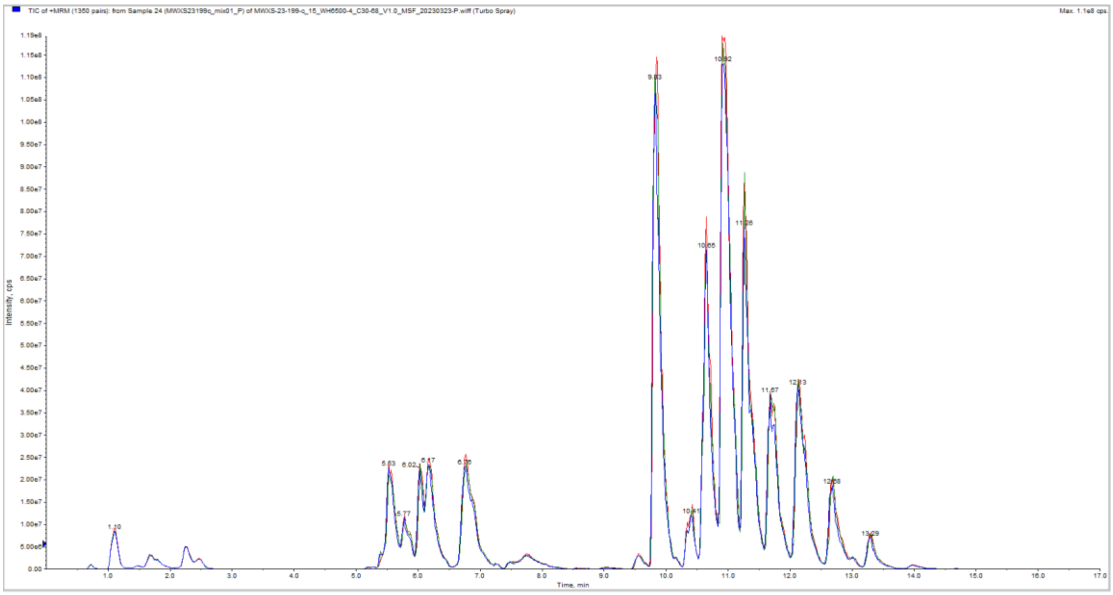

Figure S1. Overlapped total ion chromatograms (TICs) of QC samples in negative and positive ionisation modes. (A) Negative ionisation mode; (B) positive ionisation mode. The high overlap of retention time and peak intensity across all QC samples indicates good stability and repeatability of the LC-MS system during lipidomic analysis.

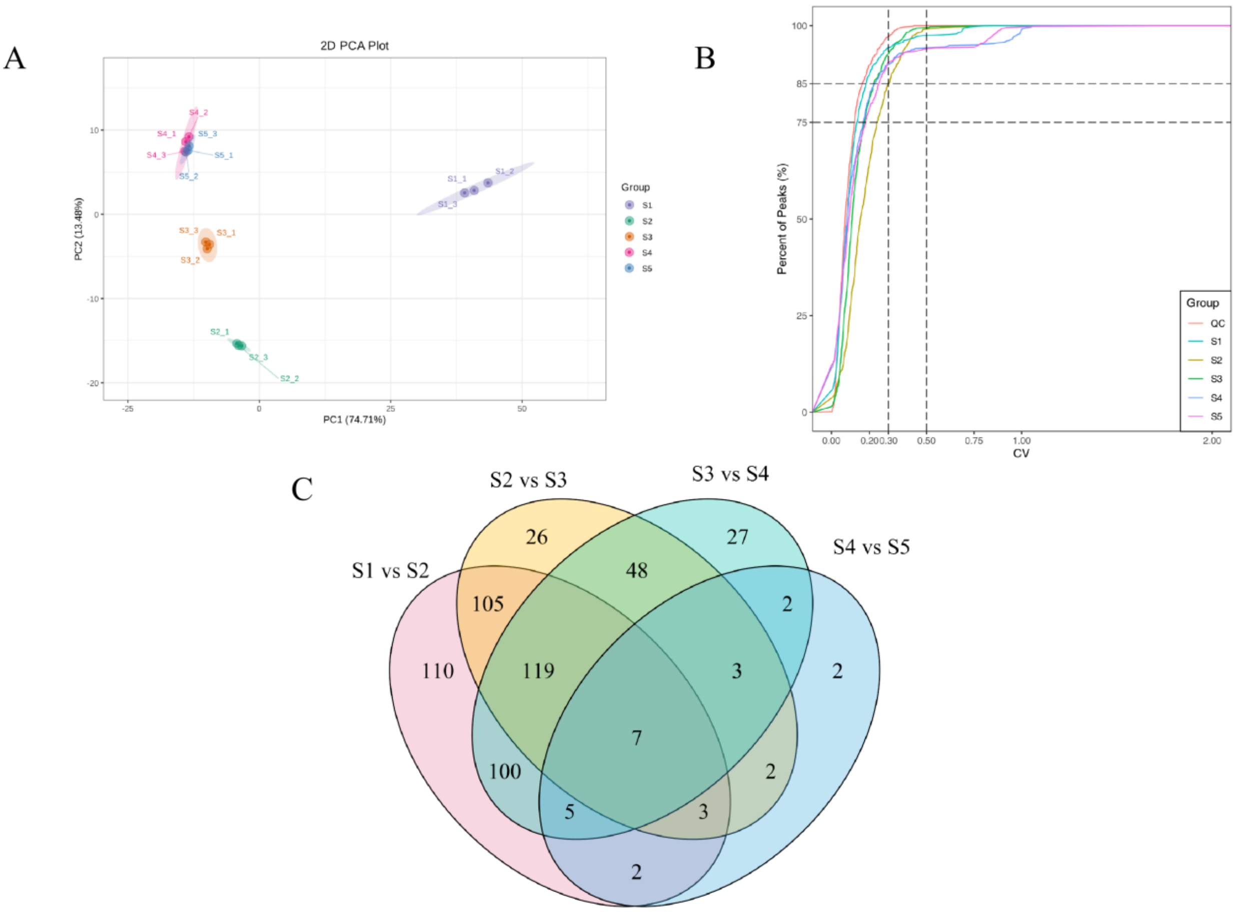

Figure S2. Overview analysis of lipidomics in *C. camphora* seed kernels. (A) Principal component analysis of QC samples. (B) CV distribution of lipid molecules. (C) Venn diagram of differential lipid molecules.

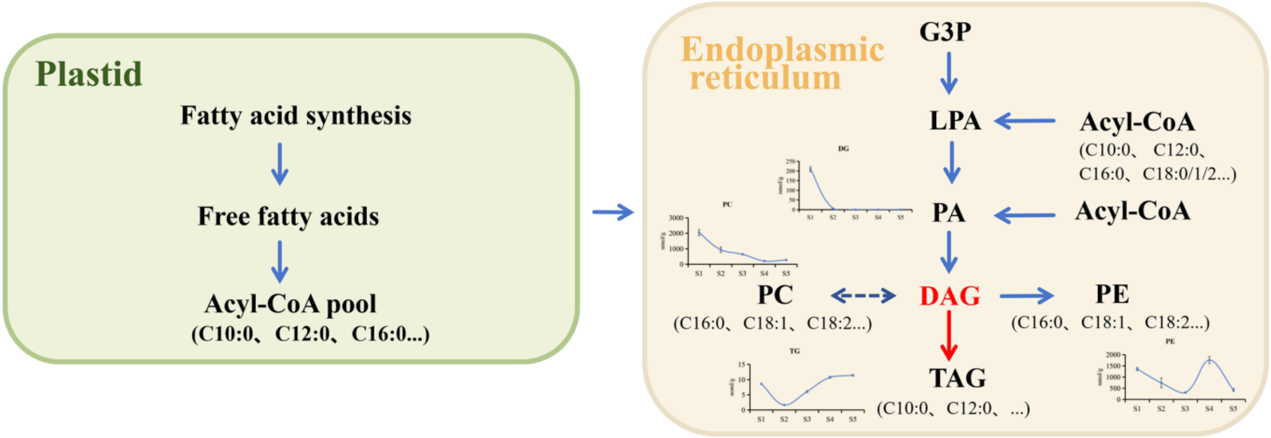

Figure S3. The Kennedy pathway of lipid metabolism in *C. camphora* seed kernels. The diagram illustrates the metabolic connections of key lipid intermediates; the dynamic changes in DG, PC, PE and TG contents across five developmental stages; and the distinct fatty acid composition characteristics of different lipid components involved in this pathway.
